# LM-QASAS: reference-free identification of antigen-specific sequences from the BCR repertoire using antibody language models

**DOI:** 10.3389/fimmu.2026.1844788

**Published:** 2026-07-30

**Authors:** Genki Masuda, Yohei Funakoshi, Shunsuke Iizumi, Kimikazu Yakushijin, Goh Ohji, Hironobu Minami, Masahito Ohue

**Affiliations:** 1 Department of Computer Science, School of Computing, Institute of Science Tokyo, Yokohama, Kanagawa, Japan; 2 Division of Medical Oncology/Hematology, Department of Medicine, Kobe University Hospital, Kobe, Hyogo, Japan; 3 Division of Infection Disease Therapeutics, Department of Microbiology and Infectious Diseases, Kobe University Hospital, Kobe, Hyogo, Japan; 4 Cancer Center, Kobe University Hospital, Kobe, Hyogo, Japan; 5 AI-Science Nexus Center, Institute of New Industry Incubation, Institute of Science Tokyo, Yokohama, Kanagawa, Japan

**Keywords:** AI and machine learning, antibody language model, B-cell receptor repertoire, SARS-CoV-2, systems immunology

## Abstract

The B-cell receptor (BCR) repertoire serves as a historical record of immunological events. However, deciphering antigen-specific sequences from this vast dataset remains a challenge, particularly for novel pathogens where prior knowledge is absent. While time-course analysis methods such as QASAS have proven effective for tracking immune responses, they rely on existing antibody databases, limiting their applicability to emerging diseases. To overcome this limitation, we introduce LM-QASAS, a reference-free computational framework that integrates antibody language models (AbLMs) with longitudinal repertoire dynamics. By mapping sequences into a high-dimensional semantic embedding space, LM-QASAS identifies clusters of functionally convergent sequences that are semantically similar and exhibit transient expansion upon immune stimulation. In a SARS-CoV-2 cohort, candidate sequences extracted by LM-QASAS were enriched for overlap with the CoV-AbDab neutralizing-antibody database relative to a random baseline. Leave-one-out cross-validation showed that these reference-free databases could reconstruct longitudinal immune-response dynamics in unseen individuals without external references. Conversely, the method showed limited sensitivity in an influenza vaccine cohort, indicating that the approach is most effective under conditions of robust, synchronized clonal expansion, such as those induced by mRNA vaccination. LM-QASAS provides a rapid, reference-free approach for monitoring humoral immunity against emerging threats.

## 1 Introduction

The adaptive immune system generates a theoretical diversity of up to 10^14^ variants via B-cell receptor (BCR) gene rearrangement, thereby addressing a vast range of pathogens (1, 2). The BCR repertoire accumulated within an individual reflects their immunological history, including infections, vaccinations, and autoimmune responses, and serves as a rich source of information for comprehensively assessing the state of the adaptive immune system (3, 4). Advances in next-generation sequencing (NGS) technologies have enabled the exhaustive analysis of this massive information, which became a key to elucidating immune dynamics during the COVID-19 pandemic (5–9).

Significant progress has been made in tracking antigen-specific responses from longitudinal BCR repertoire data in recent years. The Quantification of Antigen-Specific Antibody Sequence (QASAS) method proposed by Funakoshi et al. is a powerful framework for quantitatively visualizing responses to specific antigens by referencing known antibody sequence databases (10, 11). However, this approach relies on the existence of referenceable antibody databases. With exceptions like COVID-19, such databases are largely undeveloped for emerging infectious diseases and many intractable conditions. Thus, to realize truly versatile repertoire analysis, a technology that autonomously discovers antigen-specific sequences from the internal structure and dynamics of the repertoire data itself, without relying on external ground truth data (i.e., a reference-free approach), is indispensable.

To date, numerous computational approaches have been proposed to identify antigen-specific BCR sequences from BCR repertoire sequencing data. One major line of work is repertoire mining based on known binders (5, 12, 13). These approaches have enabled the discovery of related sequences and functionally similar antibodies by searching for sequences that belong to the same clonotype as known antigen-binding antibodies or that exhibit similarity in predicted paratopes. However, because they require known binders against the antigen of interest or sequence information enriched for antigen specificity as the starting point, their applicability is limited for emerging infectious diseases and unknown antigens for which curated databases of known antibodies are insufficient. Therefore, reference-free methods are needed that can extract antigen-responsive BCRs directly from repertoire data without relying on external antigen-specific reference sequences.

To address this challenge, reference-free computational methods that do not use known binders have also been proposed (9, 14–16). These methods exploit convergent footprints left by antigen-responsive BCRs within repertoires, including inter-individual sharing, clonality, changes in abundance, and local over-enrichment in sequence neighborhoods. For example, analyses based on public receptors or oversharing detect identical or highly similar CDR3 sequences that repeatedly appear across multiple individuals, and the statistical model of Fowler et al. integrated the observed frequency of BCR sequences, clonality, and inter-individual sharing to infer vaccine-specific BCRs. STAR identifies antigen-responsive BCR clusters by leveraging local over-enrichment in sequence neighborhoods and convergent selection in a BCR repertoire measured at a single time point. Although these studies have shown that antigen-response signals can appear as convergent patterns within repertoires, many of them rely on explicit sequence features or repertoire statistics, such as CDR3 sequence identity, edit distance, clonotype membership, sharing, or abundance. Consequently, they may not fully capture BCR groups that are distant at the sequence level but have similar antigen-recognition functions; in other words, functionally convergent BCRs.

Studies have also used neighborhood structure and density in sequence space. STAR is conceptually related to density-based approaches in that it exploits local over-enrichment in sequence neighborhoods; however, because it mainly analyzes repertoires at a single time point, it is difficult to distinguish, in a time resolved manner, whether such enrichment represents a newly induced responsive cluster after immune stimulation or a background structure that was already present before stimulation. Shingai et al. extracted infection-induced CDRH3 clusters by combining kernel density estimation in CDRH3 sequence space with subtraction of a background repertoire (17). However, this approach is based on differences from a background repertoire and does not directly use longitudinal density dynamics within the same individual, from pre-stimulation to the response peak and the contraction phase, as a criterion for antigen response. Therefore, a framework is needed that detects regions in longitudinal BCR repertoires from the same individual where local clonal density transiently increases after immune stimulation, rather than relying solely on single-time-point neighborhood enrichment or differences from background repertoires.

To address these challenges, we propose LM-QASAS (Language Model-guided QASAS), a reference free computational framework that integrates embedding representations from antibody language models (AbLMs) with longitudinal dynamics of BCR repertoires. LM-QASAS has three distinctive features. First, it does not use known binders or antigen-specific antibody databases during the extraction step. Second, by mapping each BCR sequence into an AbLM-derived semantic space, it leverages proximity based on contextual and structural features of antibody sequences beyond simple sequence identity or edit distance (18–20). In this semantic space, BCR sequences that have been selected by a common antigenic stimulus and are functionally convergent in the sense that they share similar antigen-recognition functions are expected to be positioned close to one another, even when their CDR3 sequences or clonotypes differ. Third, LM-QASAS treats pre-stimulation, response-peak, and contraction-phase BCR repertoires as time-point-specific distributions in semantic space, and extracts regions where local clonal density transiently increases at the peak as candidate antigen-responsive BCRs. Here, local density refers not to read counts, but to the degree of accumulation of BCR clones in a neighborhood of the semantic space.

From a machine-learning perspective, LM-QASAS can be positioned as density-based temporal novelty detection in AbLM semantic space (21, 22). Specifically, the pre-stimulation repertoire is regarded as an individual-specific background distribution and the response-peak repertoire as a post-intervention distribution. Regions that are low-density before immune stimulation but transiently become high-density at the peak are detected as BCR clone populations that have newly emerged in association with the antigen response. Concretely, we adopt two complementary approaches: discrete clustering using K-means (23) and k-nearest-neighbor (k-NN)-based local density estimation (24, 25). These approaches identify clone populations in semantic space that expand selectively and transiently after interventions such as vaccination. To our knowledge, this study is the first attempt to formulate the identification of antigen-responsive BCRs from longitudinal BCR repertoire data as density-based temporal novelty detection in a semantic space learned by antibody language models.

## 2 Results

### 2.1 Overview of LM-QASAS and semantic-space immune dynamics

**Figure 1:**
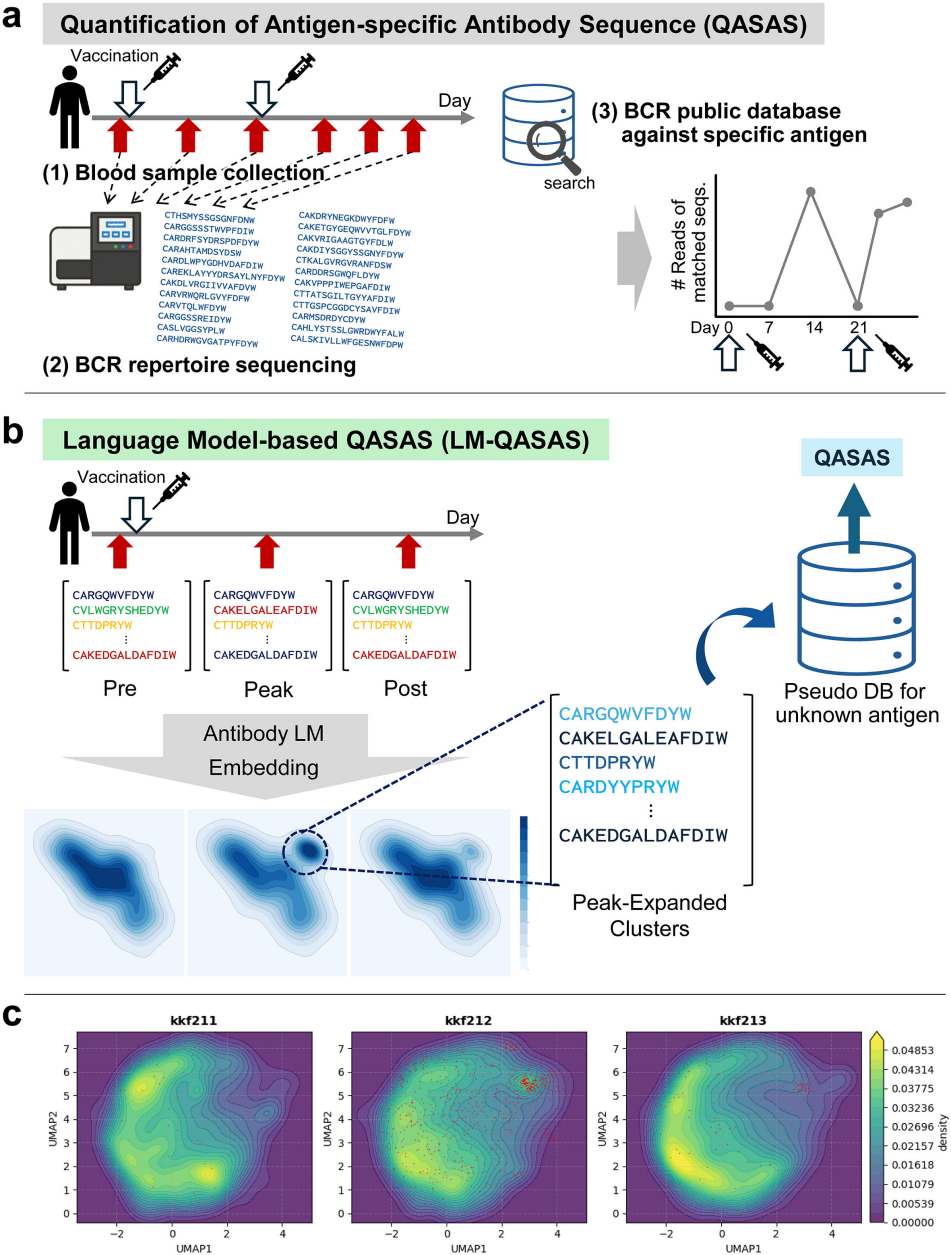
Overview of QASAS and the proposed reference-free LM-QASAS, and an example of semantic-space repertoire dynamics. **(a)** QASAS workflow. Blood samples are collected before and after SARS-CoV-2 mRNA vaccination to obtain BCR repertoire sequences. These are cross-referenced with a public neutralizing antibody database to quantify immune response dynamics based on the rise in the hit rate (overlapping rate) of sequences similar to database entries at the peak time point. **(b)** LM-QASAS workflow. Longitudinal repertoire sequences are converted into embeddings using an Antibody Language Model (AbLM), and densities at each time point are estimated. Expanded sequences are extracted from regions exhibiting increased local density at the peak followed by a decline. By using these sequences as a pseudo-database for unknown antigens, time-series analysis equivalent to QASAS is enabled without reliance on external references. **(c)** Representative semantic-space visualization. The BCR repertoire of a representative healthy mRNA vaccinee is projected into 2D space using UMAP based on AbLM embeddings, with distribution densities visualized via KDE for three time points (Left: Pre-vaccination, Center: Peak, Right: Post-vaccination). The colormap represents sequence density. Red points indicate sequences similar (≥ 85% sequence identity, i.e., length-normalized Levenshtein distance ≤ 0.15) to those in a known SARS-CoV-2 antibody database (CoV-AbDab); notably, they accumulate within the local high-density regions formed during the peak response.

**Figure 1** illustrates the analytical workflows of the existing QASAS method, which quantifies antigen-specific responses from longitudinal BCR repertoires, and our proposed LM-QASAS, a reference-database independent framework. QASAS visualizes immune responses by cross-referencing BCR sequences obtained pre- and post-vaccination against public neutralizing antibody databases and tracking the temporal changes in the hit rate (overlapping rate) of sequences similar to database entries (**Figure 1a**). In contrast, LM-QASAS extracts sequence populations that exhibit transient expansion at the peak time point within the embedding space of an antibody language model (AbLM; in this study, AbLang2 (26)), based solely on the internal dynamics of the repertoire, thereby constructing a pseudo-database for unknown antigens (**Figure 1b**). While **Figure 1b** shows an example that uses kernel density estimation (KDE) on UMAP projections for visual clarity, our actual analysis evaluated both a k-NN-based approach and a *K*-means-based approach. The latter partitions the original high-dimensional embedding space to identify candidates based on temporal fluctuations of clusters, allowing a comparative assessment of the two variants.

### 2.2 Characterization of semantic space and cluster dynamics

The LM-QASAS framework proposed in this study is grounded in the hypothesis that “antigen-specific immune responses manifest as increases in local sequence density within the semantic space constructed by an AbLM.” To validate the biological validity of this hypothesis, we analyzed longitudinal BCR repertoire data from three cohorts with distinct immune backgrounds: healthy SARS-CoV-2 mRNA vaccinees, COVID-19 convalescent patients, and post-hematopoietic stem cell transplantation (HSCT) mRNA vaccinees.

First, to visually demonstrate the fundamental principle of our method, we selected a representative subject from the healthy vaccinee cohort and analyzed the spatiotemporal dynamics of their repertoire in detail. Specifically, we vectorized all BCR sequences at three time points, pre-vaccination (Pre-vaccination), the peak of antibody titer rise (Peak), and the subsequent decline phase (Post-peak), using AbLM and projected them into a two-dimensional space using UMAP. Furthermore, we visualized the density of the sequence distribution at each time point using kernel density estimation (KDE) (**Figure 1c**). To interpret the biological meaning of this spatial arrangement, we defined sequences within the subject’s repertoire that exhibited high similarity (≥ 85% sequence identity; length-normalized Levenshtein distance ≤ 0.15) to those registered in the known SARS-CoV-2 neutralizing antibody database [CoV-AbDab (11)] as “known antigen-specific sequences” and plotted them onto the same space (**Figure 1c**, red points).

The analysis revealed that the BCR repertoire was widely dispersed throughout the semantic space at all three time points, confirming the maintenance of a diverse antibody population. However, focusing on the local density distribution revealed dramatic changes. At the pre-vaccination stage (**Figures 1c**, left), CoV-AbDab-related sequences (red points) were barely detectable, and no significant concentration in specific regions was observed. In contrast, at the peak of the immune response (**Figures 1c**, middle), a clear increase in density was confirmed in a specific local region that had been low-density prior to vaccination. Crucially, the CoV-AbDab-related sequences (red points) that appeared in large numbers at this timing were distributed extremely intensively within this newly formed high-density region. Subsequently, over time, the density of the region decreased and the number of red points declined, but we observed that a subset of sequences was retained in the same region as the peak (**Figures 1c**, right).

This visual evidence demonstrates that within the AbLM-based semantic space, antigen-specific clonal expansion is identifiable as a “local elevation in specific semantic regions” distinct from the diverse background repertoire. It strongly suggests that, by tracking changes in high-density regions, it is possible to trace the dynamics of antigen-specific antibody populations from emergence to convergence without prior reference to external databases. Note that, because UMAP distorts the underlying data structure, density estimation in this study was performed not in the UMAP space but in the high-dimensional embedding space, where both discrete and continuous local densities were estimated; UMAP was used solely for visualization.

### 2.3 Validation with known neutralizing antibody databases

The LM-QASAS framework proposed in this study was designed to extract locally formed high-density clusters in semantic space, as observed in the previous section (**Figure 1c**). Here, we evaluated two LM-QASAS variants with different density-estimation mechanisms: LM-QASAS (K-means), which is based on discrete clustering, and LM-QASAS (k-NN), which directly estimates local density in the AbLM embedding space. To isolate the contribution of the AbLM semantic space to density estimation, we also evaluated an ablation of LM-QASAS (k-NN), termed Seq-QASAS (k-NN), which applies the same k-NN density-estimation framework but replaces the AbLM-derived semantic space with a sequence space defined by pairwise CDR-H3 Levenshtein distances (27). Seq-QASAS therefore differs from LM-QASAS (k-NN) only in the representation space and is intended as an ablation of our own method rather than as an independent external baseline.

To evaluate the antigen specificity of the extracted sequences, we analyzed the top-*N* candidate sequences extracted from each subject, where *N* ∈ 50,100,300,1000, and calculated the proportion of sequences that showed high similarity to entries registered in the known neutralizing antibody database CoVAbDab (11). Because CDR-H3 sequences vary widely in length, we defined high similarity using a length-normalized Levenshtein distance ≤ 0.15 (i.e., ≥ 85% sequence identity), so that the metric does not favor shorter sequences. As additional reference-free baselines, we evaluated STAR (16), which detects antigen-responsive clones from a single peak time point through local sequence-neighborhood enrichment, and sequences randomly sampled from the repertoire at the response-peak time point (Random). The analysis included 10 subjects from three cohorts with different immune backgrounds: healthy mRNA vaccine recipients (HV, *n* = 4), convalescent COVID-19 patients (CP, *n* = 3), and mRNA vaccine recipients after hematopoietic stem cell transplantation (VN, *n* = 3). The mean overlap rates for each top-*N* threshold and for each cohort at top-300 are shown in **Table 1**.

**Table 1:** CoV-AbDab overlap rates for each method.

Method	Top-50	Top-100	Top-300	Top-1000	HV	CP	VN
LM-QASAS (K-means)	**26.40**	**24.80**	**15.60**	6.52	**30.75**	*3.56*	7.44
LM-QASAS (k-NN)	*23.00*	*20.10*	13.10	6.81	23.92	2.89	8.89
Seq-QASAS (k-NN)	17.40	17.20	*13.50*	**9.22**	*24.50*	2.44	**9.89**
STAR	21.60	19.20	12.13	*7.19*	19.75	**5.00**	*9.11*
Random	1.40	1.10	1.47	1.48	2.33	0.89	0.89

Values indicate the percentage of extracted sequences with high similarity to CoV-AbDab entries, defined as a length-normalized Levenshtein distance ≤ 0.15 (i.e., ≥ 85% sequence identity), which avoids favoring shorter CDR-H3 sequences. The top-50, top-100, top-300, and top-1000 columns show averages across all subjects (*n* = 10), whereas the HV, CP, and VN columns show cohort-wise averages at top-300. In each column, the highest value is shown in bold and the second-highest value is shown in italics. STAR is a reference-free baseline that operates on a single (peak) time point.

All three QASAS-based methods, as well as the single-time-point STAR baseline, consistently showed higher overlap than Random, indicating that antigen-responsive sequences were enriched by these reference-free approaches. However, their relative performance varied depending on the extraction depth and subject cohort.

For the top-ranked candidates, particularly at top-50, top-100, and top-300, LM-QASAS (K-means) showed the highest numerical purity among the evaluated methods. The overlap rate was 26.4% at top-50, and the mean overlap rate in the healthy mRNA vaccine recipient cohort was 30.8%. In the per-subject Wilcoxon signed-rank test comparing LM-QASAS (K-means) with Seq-QASAS (k-NN), the differences were not statistically significant at any extraction depth, with two-sided *p*-values of 0.625, 0.469, 0.496, and 0.176 for top-50, top-100, top-300, and top-1000, respectively. LM-QASAS (k-NN) also showed competitive performance and was often ranked second in these comparisons.

At top-1000 and in the post-hematopoietic stem cell transplantation vaccine recipient (VN) cohort, Seq-QASAS (k-NN) showed the highest overlap, with 9.2% at top-1000 and 9.9% in the VN cohort. In the convalescent patient (CP) cohort, the single-time-point STAR baseline showed the highest overlap, with a mean overlap rate of 5.0%. This indicates that sequence-neighborhood signals can also extract antigen-specific clones in complex immune backgrounds without relying on the AbLM semantic space, suggesting that the enrichment signal captured by these methods is not exclusively dependent on the AbLM-based representation.

Taken together, these results suggest that the relative behavior of the methods depends on extraction depth and cohort background. LM-QASAS (K-means) showed higher numerical purity among top-ranked candidates, particularly in the HV cohort, whereas sequence-neighborhood-based methods, including Seq-QASAS (k-NN) and STAR, showed higher overlap in some deeper-ranking or cohort-specific settings. Thus, the observed advantage of the AbLM-based representation was not uniform across all evaluation settings, and the mechanisms underlying these differences remain to be investigated.

### 2.4 Tracking longitudinal immune dynamics using pseudo-reference databases

The three methods evaluated in Section 2.3 were assessed using the static purity of the top-300 peak-time-point sequences against CoV-AbDab. To examine whether these sequences can also reconstruct longitudinal immune-response dynamics, we used the sequences extracted by each method as pseudo-reference databases and performed leave-one-out (LOO) tracking across the 10 SARS-CoV-2 cohort subjects.

For each held-out subject, the remaining nine subjects were used to construct method-specific pseudo-reference databases from the top-300 sequences extracted from the Peak repertoires. QASAS analysis was then applied to the repertoire at each time point of the held-out subject, and overlap with each pseudo-reference database was calculated using a Levenshtein distance threshold of ≤ 2. To compare temporal patterns across subjects and databases, overlap rates were normalized as fold-change relative to the Pre time point. CoV-AbDab and a Random database constructed from peak-time-point repertoires were used as comparators.

**Figure 2:**
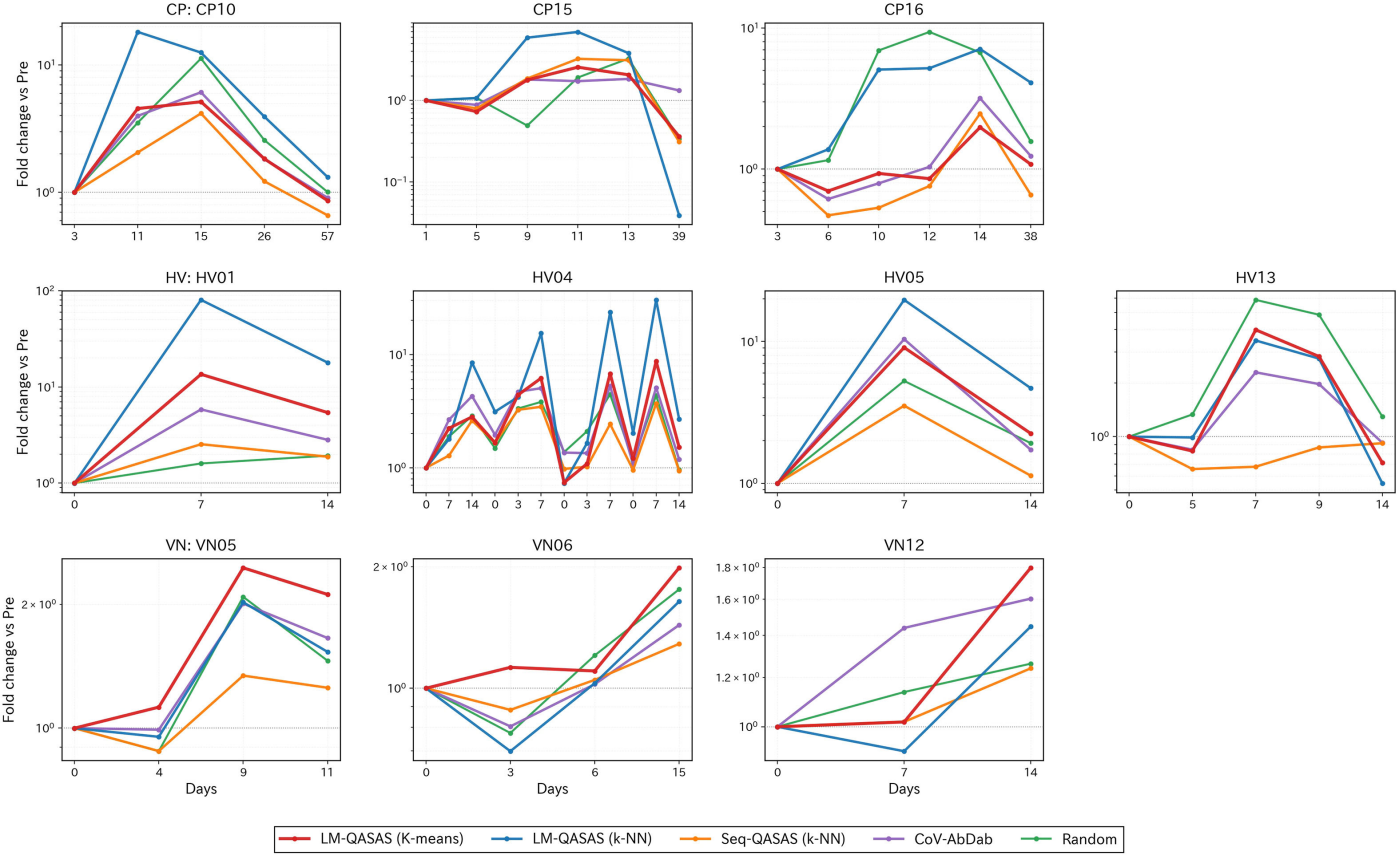
Longitudinal tracking of immune-response dynamics using pseudo-reference databases. For each held-out subject, pseudo-reference databases were constructed by LOO cross-validation from the top-300 sequences extracted from the remaining subjects. Overlap was calculated at each available time point using a Levenshtein distance threshold of ≤ 2 and normalized as fold-change relative to the Pre time point. Panels are grouped by cohort: convalescent COVID-19 patients (CP), healthy mRNA vaccine recipients (HV), and post-hematopoietic stem cell transplantation vaccine recipients (VN). Lines indicate LM-QASAS (K-means; red), LM-QASAS (k-NN; blue), Seq-QASAS (k-NN; orange), Random (green), and CoV-AbDab (purple).

As shown in **Figure 2**, all three method-based pseudo-reference databases reproduced rise, peak, and decay patterns similar to those obtained with CoV-AbDab. In the HV cohort, the response peak was consistently observed around day 7, consistent with rapid recall responses after booster vaccination (28). These results indicate that sequences extracted by each method can be used to track immune-response dynamics without relying on an external neutralizing antibody database.

The Random baseline also showed peaks in several subjects, likely reflecting public-clone-like signals shared across individuals responding to the same antigen. Therefore, the advantage of the three methods is not defined solely by fold-change magnitude, but also by their higher CoV-AbDab overlap rates at top-300 and their ability to suppress baseline overlap. Consistent with this interpretation, LM-QASAS (k-NN) showed the lowest mean Pre overlap rate among the QASAS-based methods, whereas Seq-QASAS (k-NN) showed the highest baseline overlap (**Table 2**). This suggests that AbLM-space k-NN density estimation provides stronger baseline suppression, while Levenshtein-space k-NN captures broader public sequence signals.

**Table 2:** Mean Pre-time-point overlap rates used as the baseline for fold-change normalization.

Reference database	Mean pre overlap rate (%)
LM-QASAS (K-means)	0.47
LM-QASAS (k-NN)	0.19
Seq-QASAS (k-NN)	1.36
Random	0.56
CoV-AbDab	1.60

Values indicate mean overlap rates across the 10 LOO evaluations. Overlap was defined using a Levenshtein distance threshold of ≤ 2.

In densely sampled subjects, the same framework also captured more complex dynamics. In HV04, repeated booster responses were detected across multiple vaccination rounds, whereas long-term samples from the CP cohort returned close to baseline after the response peak. Thus, pseudo-reference tracking can capture both response induction and contraction over time.

### 2.5 Evaluation on influenza vaccine cohort

To assess the applicability and limitations of LM-QASAS, we performed a similar leave-one-out cross-validation on an influenza vaccine cohort (*n* = 17), which differs from the SARS-CoV-2 cohort in both antigen and immune background. In this experiment, we used repertoire data from three time points: pre-vaccination (Day 0), post-vaccination (Day 7), and two weeks post-vaccination (Day 14).

The results for representative subjects are shown in **Supplementary Figure 1**. In contrast to the clear longitudinal tracking observed in the SARS-CoV-2 cohort, immune-response dynamics were not clearly visualized in 11 of the 17 influenza vaccine recipients, even when using LM-QASAS. Because some subjects had zero overlap at the Pre time point, fold-change normalization was not applicable in this cohort. Therefore, we evaluated the trajectories using absolute overlap rates rather than fold-change values. Although an increase in overlap rate at the expected peak time point (Day 7) was observed in some subjects, the peak was often weak and difficult to distinguish from the baseline.

To interpret these failure cases, we examined the behavior of the Random control. Whereas the Random control showed peak-like increases in the SARS-CoV-2 cohort (Section 2.4), it remained nearly flat across the three time points in the influenza cohort. We interpret this contrast as reflecting the extent of global antigen-associated repertoire fluctuation, or the effective signal-to-noise ratio of antigen-specific clones within the peripheral blood repertoire. From this perspective, the influenza vaccine cohort represents a low signal-to-noise setting, in which antigen-specific clones did not induce a sufficiently detectable density shift in repertoire space. Because LM-QASAS relies on detecting temporal increases in local clonal density, its sensitivity decreases when vaccine-induced repertoire perturbation is weak. These results suggest that LM-QASAS is most effective when antigen stimulation induces a clear and transient repertoire-level density change, whereas its performance is limited when such changes are sparse or close to background fluctuation.

## 3 Discussion

### 3.1 Summary of findings and scope of the density-based temporal detection framework

In this study, we formulated reference-free identification of antigen-responsive B-cell receptors (BCRs) as density-based temporal detection in repertoire-derived feature spaces. We evaluated this framework in both an AbLM-derived semantic space, implemented as LM-QASAS, and a Levenshtein-distance-based sequence space, implemented as Seq-QASAS. Comprehensive validation using COVID-19 cohorts showed that transient increases in local clonal density can enrich antigen-associated BCR candidates and reconstruct longitudinal immune dynamics without external antigen-specific reference databases. At the same time, the influenza vaccine cohort revealed that this density-based framework has reduced sensitivity when repertoire-level perturbation is weak and temporal density shifts are close to background fluctuation.

### 3.2 Immunological interpretation of cohort differences

A key finding of this study is that the detection performance of the density-based temporal framework varied markedly depending on subjects’ immune backgrounds (HV, CP, VN) and on the feature space used for density estimation (**Table 1**). These variations reflect both the qualitative and quantitative differences in immune responses across the cohorts and the distinct types of repertoire structure captured by the AbLM semantic space and the Levenshtein sequence space.

First, the high accuracy achieved by LM-QASAS in healthy vaccinees (HV), particularly among top-ranked candidates, can be attributed to the specific characteristics of mRNA vaccines. Previous studies have shown that, compared to conventional protein vaccines, mRNA vaccines induce more robust and persistent germinal center reactions against a single antigen (spike protein) and cause massive mobilization of plasmablasts (29, 30). This synchronous and explosive clonal expansion likely results in the formation of high-density clusters in specific regions of the AbLM semantic space, allowing the density-based extraction algorithm to function effectively.

In contrast, the reduced scores observed in convalescent patients (CP) are likely related to differences in target antigens. While mRNA vaccines target only the spike protein, natural infection exposes the immune system to the whole virus particle, inducing a broad polyclonal response not only against the spike protein but also against internal viral components such as the nucleocapsid (N) protein and the membrane (M) protein (31, 32). However, the CoV-AbDab database referenced in this study is biased toward information on spike antibodies, which are critical for preventing infection. Consequently, even if LM-QASAS successfully extracted genuine antibody clusters specific to infection, such as those targeting the N protein, it is highly likely that they were classified as mismatches with the database in this validation.

Furthermore, the lower performance in post-hematopoietic stem cell transplantation patients (VN) may reflect the structural instability of the repertoire during immune reconstitution. Post-transplant B-cell repertoires are known to exhibit lower diversity compared to healthy individuals due to insufficient supply of naive B cells and homeostatic proliferation, rendering them unstable against perturbations (33, 34). Under such conditions, vaccine-specific clonal expansion is easily buried in background noise (resulting in a reduced S/N ratio), which likely made signal extraction via density estimation difficult. However, the sequence-neighborhood methods performed relatively well in these cohorts, with Seq-QASAS leading in the VN cohort and the single-time-point STAR baseline leading in the CP cohort. This suggests that CDR-H3 sequence neighborhoods can capture public or near-public convergent signals that are not fully captured by coarse clustering in the AbLM semantic space. This complementarity indicates that future algorithmic improvements should consider how to integrate semantic-space and sequence-space density signals to amplify weak antigen-responsive patterns in complex immune backgrounds. We note that the differences in overlap rate among the non-random methods did not reach statistical significance (paired Wilcoxon signed-rank tests, *n* = 10), which likely reflects the limited sample size; these inter-method comparisons should therefore be interpreted as descriptive trends, nonetheless, the conceptual distinction between AbLM-derived and sequence-based representations makes the former a promising direction for reference-free repertoire analysis that warrants further investigation.

### 3.3 Comparison between SARS-CoV-2 and influenza responses

The discrepancy between the SARS-CoV-2 mRNA vaccine cohort and the influenza inactivated vaccine cohort (**Supplementary Figure 1**) highlights the immunological conditions required for LM-QASAS to function effectively, particularly the magnitude and synchrony of induced clonal expansion.

The SARS-CoV-2 cohort in this study included many multi-dose vaccine recipients, and, as in the influenza cohort, recall responses mobilizing pre-existing memory B cells were expected to occur. However, the two cohorts differed markedly in the extent of repertoire perturbation. mRNA vaccines are known to induce large plasmablast bursts detectable in peripheral blood and persistent germinal center reactions, owing to sustained intracellular antigen production and strong immunogenicity, even during recall responses (29, 30). This robust induction likely increased the proportion of antigen-specific clones within the total repertoire, resulting in a high signal-to-noise ratio that enabled the density-detection mechanism of LM-QASAS to operate effectively.

In contrast, seasonal influenza vaccination in adults often induces a more modest magnitude of *de novo* clonal expansion in peripheral blood than mRNA vaccination (35, 36). Although influenza vaccines can elicit rapid and antigen-specific recall responses, these responses may not produce a sufficient increase in sequence counts to alter the global structure of the peripheral blood repertoire. Consequently, the influenza cohort likely represented a low signal-to-noise setting, in which the density shift of antigen-responsive clones was too weak for LM-QASAS to detect consistently.

These results define the practical applicability of LM-QASAS. The method is expected to be most reliable when antigen-specific B-cell clones undergo robust and synchronized expansion in peripheral blood, when the sampling schedule captures both the expansion peak and the subsequent contraction phase, and when the immune stimulus is relatively homogeneous. Conversely, caution is required when applying LM-QASAS to multivalent vaccines, weak recall responses, or cohorts with heterogeneous prior immune histories. In the influenza vaccine cohort, the multivalent nature of seasonal influenza vaccines and the strong influence of pre-existing memory responses may have distributed the response across multiple strain-specific or cross-reactive B-cell populations, thereby reducing the local density changes detectable by LM-QASAS. Thus, LM-QASAS is best suited for immune perturbations that induce clear, transient, and repertoire-level density changes, such as those observed after strong immune stimulation by mRNA vaccination.

A broader validation of these conclusions across a wider range of antigens and immune responses is an important direction for future work, and will become feasible as well-curated longitudinal BCR repertoire datasets covering the pre-stimulation, peak, and contraction phases become available for antigens beyond SARS-CoV-2 and influenza.

## 4 Conclusion

This study demonstrates that density-based temporal analysis of longitudinal BCR repertoires can identify antigen-responsive BCR candidates without relying on external antigen-specific reference databases. By implementing this framework in both an AbLM-derived semantic space (LM-QASAS) and a Levenshteindistance-based sequence space (Seq-QASAS), we showed that transient increases in local clonal density provide a useful reference-free signal of antigen-associated repertoire dynamics.

Our comparative analysis further indicates that the choice of representation space influences which immune-response patterns are captured. LM-QASAS, particularly the K-means variant, showed the highest numerical purity among top-ranked candidates in healthy SARS-CoV-2 mRNA vaccinees, performing comparably to or above a reference-free single-time-point baseline (STAR). Although the differences among the non-random methods did not reach statistical significance, these results suggest that the AbLM semantic space provides a promising alternative representation for capturing functionally convergent BCR populations under conditions of strong and synchronized clonal expansion, and one that warrants further investigation. In contrast, Seq-QASAS provided complementary sensitivity in broader rankings and in more complex immune backgrounds, indicating that Levenshtein sequence-space neighborhoods can capture public or near-public CDR-H3-level signals that may not always form clear clusters in the AbLM semantic space.

Taken together, these results position LM-QASAS and Seq-QASAS as complementary implementations of a broader density-based temporal detection framework. Although sensitivity remains limited in low signal-to-noise settings, such as the influenza vaccine cohort, this framework provides a reference-free approach for monitoring humoral immune dynamics, particularly when antigenic stimulation induces a sufficiently strong and temporally coordinated repertoire perturbation.

## 5 Materials and methods

### 5.1 Study datasets and ethics

This study was approved by the Medical Research Ethics Committee of the Kobe University Hospital Ethics Committee (Protocol no. B240101) and was conducted in accordance with the Declaration of Helsinki and relevant ethical guidelines. All samples were collected at Kobe University Hospital between July 2020 and January 2025. Written informed consent was obtained from all participants prior to participation.

For the analysis, we used longitudinal BCR repertoire data from SARS-CoV-2-related cohorts obtained in a previous study by Funakoshi et al. (10), as well as separately obtained data from an influenza vaccine cohort. The breakdown of the SARS-CoV-2-related cohorts, as shown in **Table 3**, consists of COVID-19 convalescent patients (CP, *n* = 3), healthy volunteers vaccinated with mRNA vaccine (Pfizer BNT162b2) (HV, *n* = 4), and patients vaccinated with the same vaccine after hematopoietic stem cell transplantation (VN, *n* = 3). Peripheral blood samples were collected longitudinally from each subject for up to two months, centering around two weeks after infection or vaccination (peak time). For the influenza vaccine cohort, peripheral blood samples were obtained from 17 healthy individuals vaccinated with seasonal influenza vaccine at pre-vaccination (Day 0), 7 days post-vaccination (Day 7), and 14 days post-vaccination (Day 14). Importantly, BCR repertoire sequences derived from the SARS-CoV-2-related cohorts analyzed in this study were not included in CoV-AbDab, which was used for downstream evaluation. Thus, CoV-AbDab served as an external reference database independent of the study cohorts.

**Table 3:** Details of the SARS-CoV-2 Cohorts used in this study.

Group ID	Description	*n*	Sampling period
CP	COVID-19 Convalescent Patients Infected with SARS-CoV-2	3	Aug. – Dec. 2020
HV	Healthy Volunteers Vaccinated with mRNA BNT162b2, Naive to SARS-CoV-2	4	May – Dec. 2021
VN	Vaccinated Patients post-Hematopoietic Stem Cell Transplantation	3	Dec. 2022 – Jan. 2024

### 5.2 BCR library preparation and sequencing

BCR repertoire analysis was performed using unbiased next-generation sequencing technology developed by Repertoire Genesis Inc. (37). Analysis of the SARS-CoV-2-related cohorts (HV, CP, VN) was performed by Repertoire Genesis Inc., while analysis of the influenza vaccine cohort was performed by Takara Bio Inc.; however, the basic experimental protocols and analysis pipelines are common.

Specifically, cDNA was synthesized from total RNA using SuperScript III Reverse Transcriptase (Invitrogen) and polyT18 primers (BSL-18E). After synthesizing double-stranded (ds) cDNA, a P10EA/P20EA dsDNA adaptor was ligated and digested with NotI restriction enzyme. Subsequently, nested PCR was performed using KAPA HiFi DNA Polymerase (Kapa Biosystems) with IgG constant region-specific primers (CG1 and CG2) and an adaptor primer (P20EA). Amplicon libraries were prepared by a second PCR amplification using P22EA-ST1 and CG-ST1-R primers, and index (barcode) sequences were added by amplification using the Nextera XT Index Kit v2 Set A (Illumina). Sequencing was performed using the Illumina MiSeq paired-end platform (2×300 bp).

### 5.3 Sequence data processing

Repertoire analysis software proprietary developed by Repertoire Genesis Inc. was used for processing the obtained sequence data. Each sequence was assigned V, D, and J genes and CDRH3 regions were identified based on identity with reference sequences from the international ImMunoGeneTics information system^®^(IMGT) database (38) (https://www.imgt.org). Data analysis for the influenza cohort was also processed at Takara Bio Inc. using equivalent software. Strict filtering criteria were applied to ensure the reliability of the analysis. Specifically, only sequences containing no stop codons and that were in-frame were retained. In addition, only sequences with functional V, D, and J genes were included in the analysis; sequences determined to be ORFs or pseudogenes were excluded. Furthermore, sequences with a CDRH3 amino acid length of less than 5 were excluded from the analysis to eliminate alignment errors and non-specific fragments.

### 5.4 Definition of unique clones and input features

In this study, sequence groups in which all four elements, V gene, J gene, CDRH3 amino acid sequence, and isotype, matched were defined as the same BCR clone. Therefore, even if the CDRH3 amino acid sequences are identical, they are treated as separate unique clones if other elements such as V gene or isotype differ. In the LM-QASAS analysis, each of these unique clones is treated as a single input data point. For the calculation of embedding representations by AbLM, only the CDRH3 amino acid sequence of each clone is used; V/J gene and isotype information are not used for vector generation. Furthermore, since this method focuses on the density distribution of sequences in the semantic space, read count information for each unique clone was intentionally excluded from the analysis. By treating all unique clones as equivalent points, this approach eliminates bias caused by high-frequency clones and aims to detect variants that are functionally important despite their low frequency.

### 5.5 LM-QASAS algorithm

LM-QASAS is designed to identify antigen-specific clusters based on temporal changes in local density within the semantic space. In this study, we implemented two complementary variants that differ in how local density is estimated: (i) LM-QASAS (K-means), which is based on discrete clustering, and (ii) LM-QASAS (k-NN), which directly estimates local density in a high-dimensional embedding space. Both variants quantify a transient increase in local density at the response-peak time point after vaccination or infection using a common mathematical framework.

#### 5.5.1 LM-QASAS (K-means)

For each subject, unique sequences from all three time points (Pre, Peak, and Post) were pooled, and their embedding vectors were partitioned into *K* clusters using K-means clustering. For each cluster *C_i_*, we calculated a score, *S*_K–means_, that integrates the increase from Pre to Peak and the decrease from Peak to Post, as defined in Equation 1:

(1) $$S_{\text{K}-\text{means}}\left(C_{i}\right)=\frac{n_{peak}^{\left(i\right)}}{n_{pre}^{\left(i\right)}+\epsilon }+\frac{n_{peak}^{\left(i\right)}}{n_{post}^{\left(i\right)}+\epsilon },$$

where $n_{t}^{\left(i\right)}$ denotes the number of sequences in cluster *C_i_* at time point *t*, and *ϵ* is a pseudocount used to avoid division by zero. Finally, among the sequences present in the Peak repertoire, those belonging to high-scoring clusters were extracted as candidate antigen-specific sequences.

#### 5.5.2 LM-QASAS (k-NN)

To estimate local density while preserving continuous semantic proximity in the AbLM embedding space (480 dimensions), we calculated, for each sequence *x* in the Peak repertoire, the distance to its *k*-th nearest neighbor, *r_k,t_*(*x*), in the point set *R_t_* at each time point *t* using cosine distance. We then used the quantity defined in Equation 2 as a proxy for local density:

(2) $${\hat{d}}_{t}\left(x\right)\propto \frac{1}{r_{k,t}\left(x\right)}.$$

The k-NN-based score was defined in the same form as the K-means-based score, as shown in Equation 3:

(3) $$S_{\text{k}-\text{NN}}\left(x\right)=\frac{{\hat{d}}_{peak}\left(x\right)}{{\hat{d}}_{pre}\left(x\right)+\epsilon }+\frac{{\hat{d}}_{peak}\left(x\right)}{{\hat{d}}_{post}\left(x\right)+\epsilon }.$$

This score ranks sequences highly when their neighborhood density in semantic space sharply increases at the Peak time point and subsequently decreases at the Post time point.

#### 5.5.3 Selection of key parameters

The key parameters controlling the granularity of local-density estimation, namely the number of clusters *K* for LM-QASAS (K-means) and the number of neighbors *k* for LM-QASAS (k-NN), were determined by grid search. The evaluation metric was the CoV-AbDab overlap rate of the top-300 extracted sequences across all 10 subjects in the SARS-CoV-2 cohort, using the length-normalized similarity criterion (≥ 85% sequence identity) defined in Section 2.3. The search ranges for *K* and *k* are summarized in **Supplementary Table 1**, and the sensitivity analyses are shown in **Supplementary Tables 2, S3**. In all analyses presented in this paper, we used *K* = 500 for LM-QASAS (K-means) and *k* = 200 for LM-QASAS (k-NN), which maximized the mean top-300 CoV-AbDab overlap rates for the K-means and k-NN variants, respectively.

### 5.6 STAR baseline

As a reference-free baseline that does not use longitudinal information, we implemented STAR (Single Time-point Antibody Recognition) (16). For each subject, STAR analyzes only the response-peak repertoire (the same peak time point used by LM-QASAS) and scores each amino-acid CDR-H3 by the local density of its single-substitution neighborhood: the score is the fraction of the repertoire that lies within one amino-acid substitution (Hamming distance ≤ 1) of the sequence, where each amino-acid CDR-H3 is weighted by the number of distinct V(D)J clonotypes that encode it. Sequences are then ranked by this score and the top-*N* are extracted as candidates, as for the other methods. We used the fast variant of STAR; the generation-probability-based full variant was not used. Like LM-QASAS, STAR is reference-free and uses no external antibody database during extraction; unlike LM-QASAS, it relies on a single time point and therefore does not model longitudinal density dynamics.

## Data Availability

The data used in this study consist of human B-cell receptor repertoire sequences derived from hospital patients. These data contain sensitive information and are subject to ethical and legal restrictions approved by the institutional review boards of the participating medical institutions. Therefore, the raw sequencing data cannot be made publicly available. Access to the data may be considered upon reasonable request to the corresponding authors, subject to institutional approval and applicable regulations.
