# Supplementary material for: LM-QASAS: reference-free identification of antigen-specific sequences from the BCR repertoire using antibody language models
Source: Front Immunol. 2026 Jul 30;17:1844788. doi: 10.3389/fimmu.2026.1844788 (PMC13468803; doi:10.3389/fimmu.2026.1844788)
Supplement: Supplementary file 1 [file DataSheet1.pdf]

frontiers

Supplementary Materials

LM-QASAS: Reference-free identification of antigen-specific sequences from the BCR repertoire using antibody language models

Genki Masuda [1,†], Yohei Funakoshi [2,†,*], Shunsuke Iizumi [1,†], Kimikazu Yakushijin [2], Goh Ohji [3], Hironobu Minami [2], Masahito Ohue [1,*]

[1] *Department of Computer Science, School of Computing, Institute of Science Tokyo, 4259-G3-56 Nagatsutacho, Midori-ku, Yokohama City, 226-8501, Kanagawa, Japan*
[2] *Division of Medical Oncology/Hematology, Department of Medicine, Kobe University Hospital, 7-5-2 Kusunoki-cho, Chuo-ku, Kobe City, 650-0017, Hyogo, Japan*
[3] *Division of Infection Disease Therapeutics, Department of Microbiology and Infectious Diseases, Kobe University Hospital, 7-5-2 Kusunoki-cho, Chuo-ku, Kobe City, 650-0017, Hyogo, Japan*
[†] *These authors contributed equally to this work*

Correspondence*:
Yohei Funakoshi, Masahito Ohue
yohei@med.kobe-u.ac.jp, ohue@comp.isct.ac.jp

A. SENSITIVITY ANALYSIS OF KEY PARAMETERS

Search design

The two LM-QASAS variants, LM-QASAS (K-means) and LM-QASAS (k-NN), both quantify a local density increase at the Peak time point using a common mathematical framework. Each variant has a key parameter that controls the granularity of local-density estimation: the number of clusters K for K-means and the number of neighbors k for k-NN. These parameters define conceptually corresponding axes, as both determine the local scale used for density estimation.

The evaluation metric was the mean CoV-AbDab overlap rate of the top-N extracted sequences across all 10 subjects in the SARS-CoV-2 cohort, using the length-normalized similarity criterion ($\geq 85\%$ sequence identity), as in Main Table 1. The cohort consisted of healthy mRNA vaccine recipients (HV, $n = 4$), convalescent COVID-19 patients (CP, $n = 3$), and mRNA vaccine recipients after hematopoietic stem cell transplantation (VN, $n = 3$). The search ranges for the two key parameters are summarized in Table S1.

Table S1. Search ranges for the key parameters of the two LM-QASAS variants.

Method	Key parameter	Search range
K-means	Number of clusters K	$\{50, 100, 200, 300, 500, 1000\}$
k-NN	Number of neighbors k	$\{5, 10, 20, 50, 100, 200, 300\}$

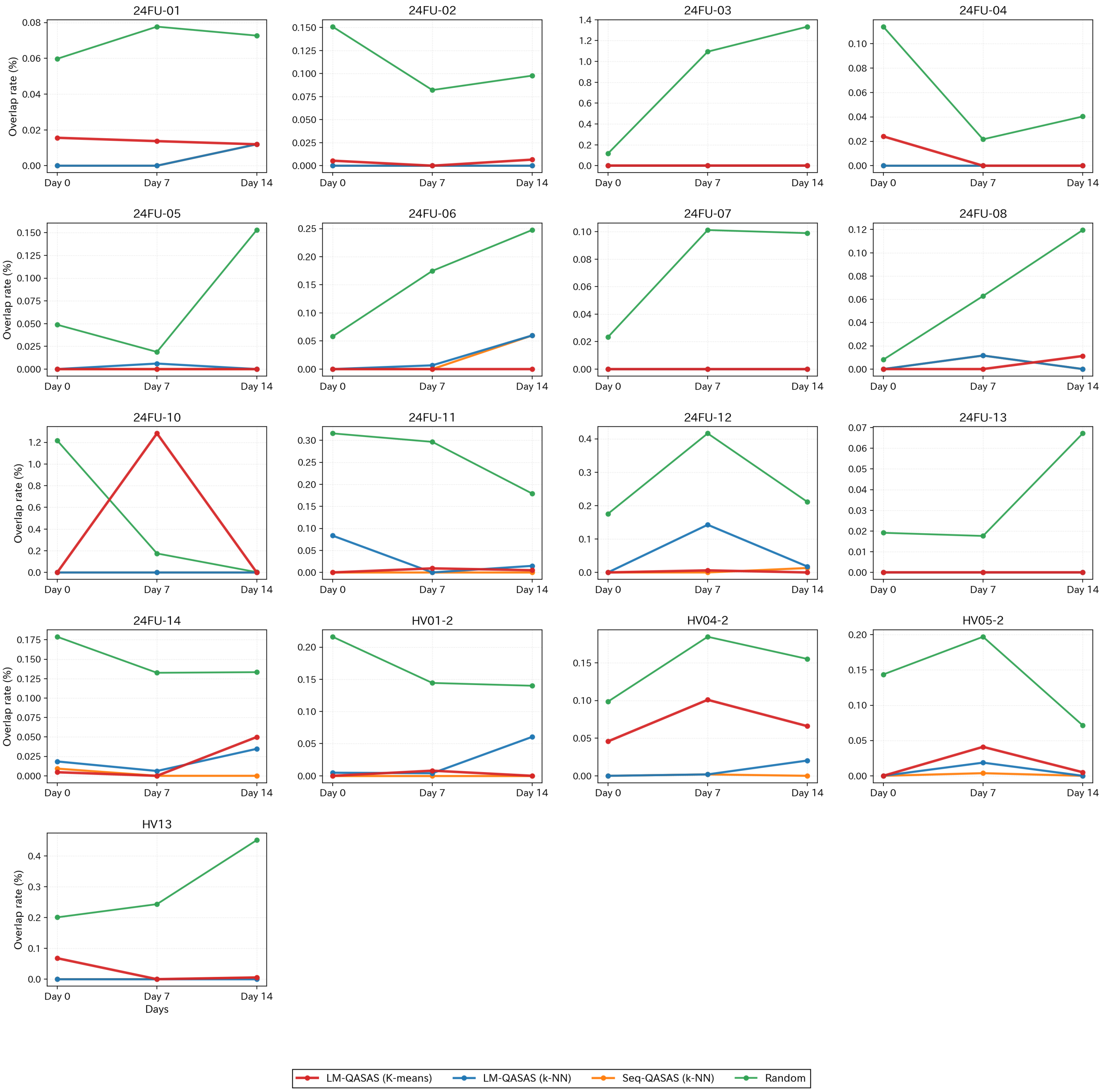

Figure S1. Longitudinal tracking of immune-response dynamics in the influenza vaccine cohort using pseudo-reference databases. The analysis was performed using the same leave-one-out pseudo-reference tracking framework as in Main Figure 2. For each held-out subject, pseudo-reference databases were constructed from the top-300 sequences extracted from the remaining subjects, and overlap was calculated at Day 0, Day 7, and Day 14 using a Levenshtein distance threshold of ≤ 2. Because some subjects had zero overlap at Day 0, trajectories are shown as absolute overlap rates rather than fold-change values. Lines indicate LM-QASAS (K-means; red), LM-QASAS (k-NN; blue), Seq-QASAS (k-NN; orange), and Random (green), following the same color scheme as Main Figure 2.

Dependence of the K-means variant on K

The sensitivity of the K-means variant to the key parameter K is shown in Table S2.

Table S2. Dependence of the K-means variant on the number of clusters K. Values indicate the mean CoV-AbDab overlap rate (%) for each top-N threshold and the cohort-wise mean overlap rate at top-300.

K	top-50	top-100	top-300	top-1000	HV	CP	VN
50	11.8	10.4	7.1	4.2	14.0	3.3	1.6
100	<u>17.6</u>	14.0	8.3	4.7	14.6	**4.2**	4.0
200	15.8	<u>16.0</u>	9.7	4.9	17.8	2.1	6.7
300	17.0	14.0	10.3	5.9	22.0	1.3	3.8
500	**26.4**	**24.8**	**15.6**	<u>6.5</u>	**30.8**	<u>3.6</u>	<u>7.4</u>
1000	5.4	10.4	<u>13.1</u>	**7.4**	<u>23.4</u>	0.7	**11.8**

The number of clusters K was a dominant parameter. The mean top-300 overlap rate generally increased from $K = 50$ to $K = 500$ (7.1% to 15.6%), reaching its maximum at $K = 500$. At $K = 1000$, top-ranked precision markedly decreased, with the top-50 overlap rate dropping from 26.4% to 5.4%. In contrast, the top-1000 overlap rate and the VN cohort average exceeded those at $K = 500$, suggesting that a finer granularity may be beneficial for detecting lower-ranked candidates or responses in immunologically unstable cohorts. In the main analyses, we therefore used $K = 500$, which maximized the mean top-300 overlap rate.

Dependence of the k-NN variant on k

The sensitivity of the k-NN variant to the key parameter k is shown in Table S3.

Table S3. Dependence of the k-NN variant on the number of neighbors k. Values indicate the mean CoV-AbDab overlap rate (%) for each top-N threshold and the cohort-wise mean overlap rate at top-300.

k	top-50	top-100	top-300	top-1000	HV	CP	VN
5	0.6	1.5	1.4	1.6	2.7	0.2	0.9
10	1.8	1.9	2.7	2.8	3.8	0.1	3.8
20	5.2	4.9	4.4	4.0	5.2	0.1	7.6
50	11.2	9.1	7.5	6.4	9.3	0.4	**12.0**
100	18.6	16.1	10.8	6.7	16.4	2.1	<u>11.9</u>
200	**23.0**	**20.1**	**13.1**	**6.8**	<u>23.9</u>	**2.9**	8.9
300	<u>20.4</u>	<u>18.6</u>	<u>12.8</u>	<u>6.8</u>	**24.3**	<u>2.9</u>	7.3

The number of neighbors k was also a dominant parameter. The mean top-300 overlap rate monotonically increased from 1.4% at $k = 5$ to 13.1% at $k = 200$, and then slightly decreased at $k = 300$. This trend suggests that excessively small k values make density estimation unstable and sensitive to noise, whereas excessively large k values make the neighborhood structures used for Peak and Pre/Post more similar, thereby attenuating the density ratio. In the main analyses, we therefore used $k = 200$, which maximized the mean top-300 overlap rate.

B. TOP-30 SCORING SEQUENCES EXTRACTED BY LM-QASAS

To enable independent experimental validation, the top-30 scoring sequences extracted by each LM-QASAS variant for each subject are provided as a supplementary CSV file available from the journal website. The CSV file contains approximately 600 candidate sequences, corresponding to the top-30 sequences extracted by two methods from 10 subjects. For each entry, the file includes the subject ID, method name, rank, CDR-H3 amino acid sequence, and associated score.
